# Incidentally recognized COVID-19 pneumonia in routine oncologic ^18^F-FDG PET/CT examinations: a local experience during pandemic era

**DOI:** 10.1186/s43055-020-00333-9

**Published:** 2020-11-04

**Authors:** Susan Adil Ali, Moustafa Mahmoud Abdelkawi

**Affiliations:** 1 Radiodiagnosis Department, Ain Shams University, Cairo, Egypt; 2 Radiodiagnosis Department, Helwan University, Cairo, Egypt

**Keywords:** COVID-19, ^18^F-FDG PET/CT, Asymptomatic, Pandemic

## Abstract

**Background:**

CT chest findings of COVID-19 pneumonia can be detected before the clinical symptoms become evident in many cases. In this work, we presented our experience in incidental detection of COVID-19-associated pneumonia in asymptomatic patients coming for routine oncologic ^18^F-FDG PET/CT examinations during the COVID-19 pandemic, which contribute in the detection of the affected patients early to be isolated and properly managed. We reported the cases with incidental finding of COVID-19 pneumonia among 764 asymptomatic patients who were referred for whole-body ^18^F-FDG PET/CT examinations for routine oncologic indications in the period between 15 March and 15 June 2020, and RT-PCR testing for them was requested for confirmation.

**Results:**

Among the 764 scanned patients, we had recognized 87 patients (11.3%) having features of COVID-19 pneumonia. RT-PCR testing of them confirmed COVID-19 infection in 78 cases, yet 3 were negative and no RT-PCR testing was performed in 6 cases (only isolated and carefully monitored).

**Conclusion:**

^18^F-FDG PET/CT is sensitive for early COVID-19 detection, even in asymptomatic patients that guide proper management and also highlight the key role of a radiologist and the importance of applying safety measures in clinical services during the pandemic to minimize the spread of infection.

## Background

COVID-19 is a novel coronavirus of zoonotic origin that occurred initially in Wuhan, China, during December 2019, and then spread worldwide, becoming a pandemic [1–3]. The most common manifestations of COVID-19 clinically include fever, cough, dyspnea, myalgia, and fatigue. However, others may initially or entirely remain asymptomatic and significantly propagate infection in the community [4]. Although reverse transcriptase-polymerase chain reaction (RT-PCR) from nasopharyngeal swabs is considered the gold standard for the diagnosis of COVID-19 infection, yet false negative results are not uncommon (sensitivity 60–70%) [5, 6]. Therefore, another examination with higher accuracy is needed to improve the detection of affected patients during the pandemic. Radiology can be helpful by identifying the pattern of pulmonary involvement of COVID-19 in chest radiologic studies [7]. CT chest findings, which consist of multiple mainly subpleural ground-glass opacities (GGOs) or consolidations, typically describe COVID-19-associated pneumonia [8, 9]. While ^18^F-FDG PET/CT examinations are not used in the primary diagnosis of COVID-19 infection, yet incidental detection of patterns of COVID-19 pneumonia in asymptomatic but infected cases referred for ^18^F-FDG PET/CT scans for other oncologic indications may necessitate further management for the patients and the health care workers [10]. This also illustrates the importance of strictly applying the recommended containing measures to limit the spread of viral infection by accurate cleaning and sterilization of the imaging room at the end of each procedure and providing adequate personal protective equipment (PPE) to all patients and to the staff members [11].

The purpose of the present study was to highlight the important role of detection of occasional radiologic findings in this historic period by presenting our local experience in incidental detection of COVID-19-associated pneumonia in our routine ^18^F-FDG PET/CT examinations for asymptomatic oncologic patients, as they are considered a hidden source of the spread of infection in the community and also affect further management for these patients.

## Methods

### Patients

This cross-sectional study was conducted in the period from 15 March to 15 June 2020 and included 764 asymptomatic patients referred for whole-body ^18^F-FDG PET/CT scanning for routine oncologic indications. Two hundred ninety-six patients came for initial assessment of recently diagnosed primary neoplasia and 468 came for a follow-up to assess the therapeutic response or for detection of recurrence. The cases with incidental findings of COVID-19 pneumonia were reported, and RT-PCR testing was requested for confirmation. The ^18^F-FDG PET/CT studies were performed after obtaining informed written consent from the patients, and the study protocol was approved by the institutional research ethics committee.

### ^18^F-FDG PET/CT technique

Combined PET/CT scan using a hybrid PET/CT system (PHILIPS; Ingenuity TF 128 PET/CT scanner; USA) was performed for all patients. The rules of patient preparation were followed strictly. Complete fasting except for glucose-free hydration for about 6–8 h before the study and their blood glucose value was kept less than 200 mg/dl at the time of the tracer injection. The scan was performed about 60 min after IV injection of 0.1 mCi of ^18^F-FDG/kg adjusted according to patient’s weight. The study was done with the patient in a supine position on the whole body from the skull base down to the mid thighs.

Initial PET scan with several bed positions (5–7) was performed and each with approximately 15-cm axial field of view per bed position with 4-mm in-plane spatial resolution and was covering the same field of view of the CT. The time of acquisition emission data was about 2 min for each bed position and in time range between 13 and 17 min.

A diagnostic contrast-enhanced CT transmission scan was done immediately after PET scanning covering the identical transverse field of view using the following parameters: 350 mA, 120 kV, 0.5-s tube rotation time,5-mm slice thickness, and 8-mm table feed. An iodinated non-ionic contrast agent (Omnipause 350) was administrated IV (100 ml) using an injector, with an injection flow of 5 ml/s just before the beginning of the scan. The lung CT images were reconstructed using a 512 × 512 matrix and iterative reconstruction, 1.25 mm (D-690) or 2.5 mm (D-STE) slice thickness and 1.25 mm interval, lung filter with a window setting with 1600 Hounsfield units (HU) window width and 480 HU window level.

A PHILIPS workstation was used to view all CT, PET, and PET/CT images, and they were reconstructed in multi-planar reformation and viewed in different planes for all as well as “3D maximum intensity projection (MIP) images” PET image in a video mode.

A teamwork, including a nuclear medicine physician and a radiologist (of 10 years of experience), had reviewed and interpreted the PET, CT, and fused PET/CT images.

The collected data were coded, tabulated, and analyzed; categorical variables (qualitative data) were presented as number and percentage; and descriptive analysis was done for numerical (quantitative) data.

## Results

Among the 764 patients (413 males and 351 females) who were included in the study, 87 patients ( about 11.4%) show incidental CT features of COVID-19-associated pneumonia that are FDG avid on the corresponding PET images (ranged between 4.3 and 12.6 SUVmax).

**Fig. 1:**
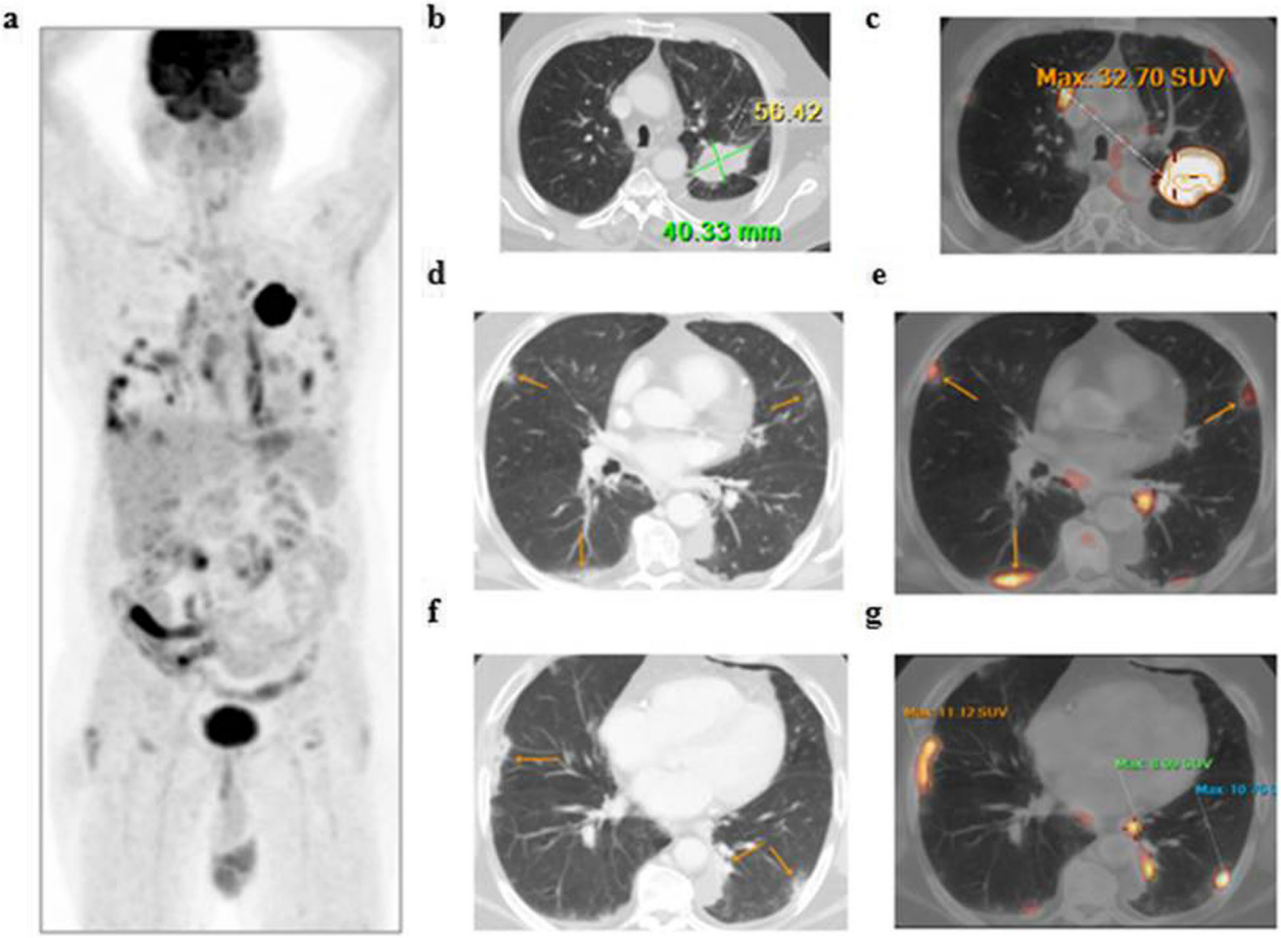
A 55-year-old male patient, presented with recently diagnosed left lung cancer and referred for initial PET/CT evaluation. **a** The whole-body MIP image shows an FDG avid upper left lung lesion with other multiple bilateral pulmonary metabolically active areas. Axial lung window CT (**b**) and corresponding fused PET/CT (**c**) images revealed an FDG avid left lung upper lobar speculated primary neoplastic mass (achieving 32.7 SUVmax), while axial CT images (**d**, **f**) revealed multiple bilateral subpleural variable-sized ground-glass opacities and patchy consolidation that show variable FDG avidity in corresponding fused PET/CT images (**e, g**) achieving up to 11.5 SUVmax

**Fig. 2:**
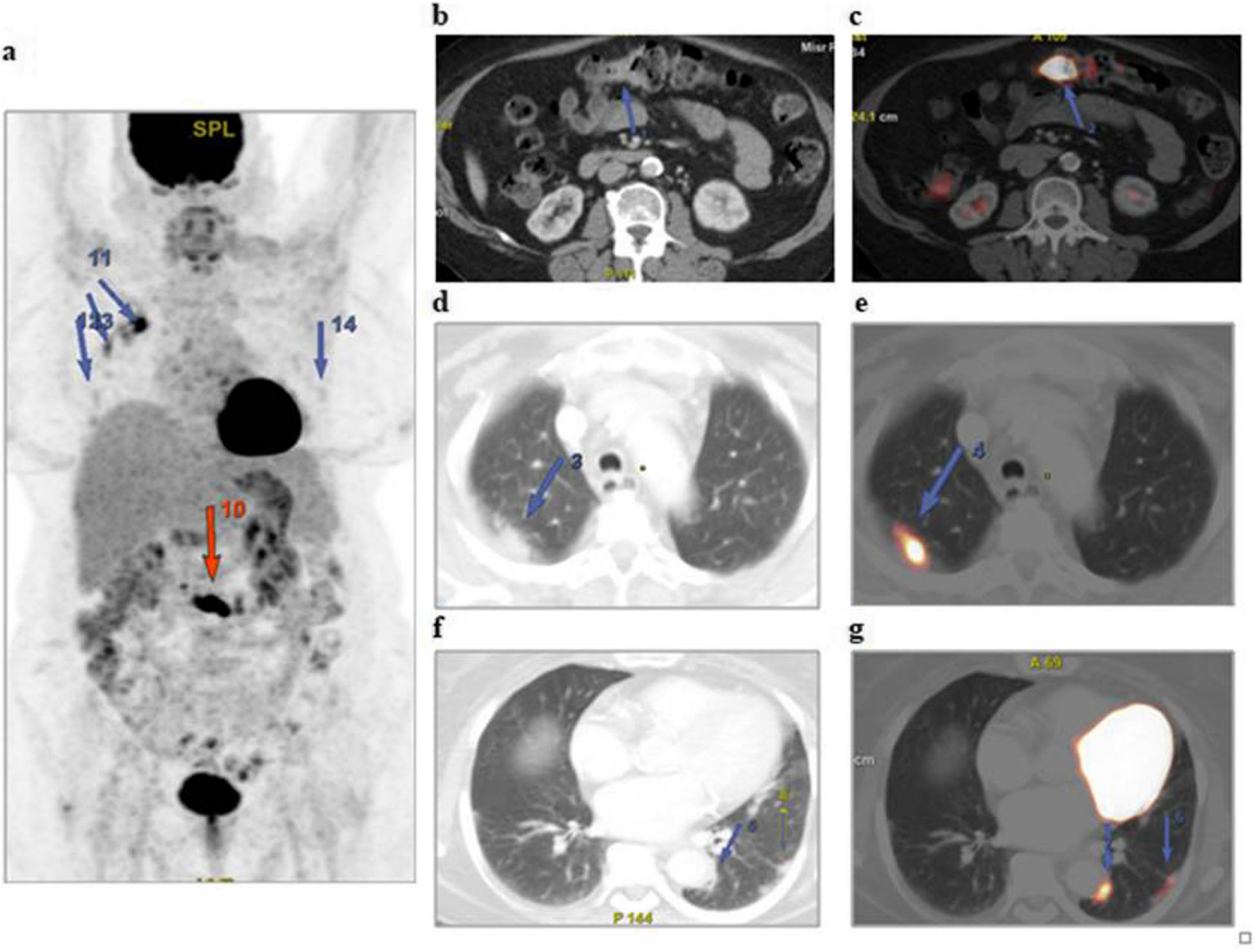
A 70-year-old female patient, presented with recently diagnosed cancer colon (adenocarcinoma grade II) and referred for initial PET CT evaluation. The whole-body MIP image (**a**) shows FDG avid lesion at the mid transverse colon (red arrow) with other bilateral pulmonary active lesions of variable FDG uptake (blue arrows). Axial abdominal CT and corresponding fused PET/CT images (**b**, **c**) revealed a short segment of FDG avid irregular circumferential malignant soft tissue mural thickening at the mid transverse colon. Axial lung window CT images (**d**, **f**) revealed bilateral subpleural ground-glass opacities and consolidations that show variable FDG avidity on corresponding fused PET/CT images (**e**, **g**) achieving up to 6.9 SUVmax

**Fig. 3:**
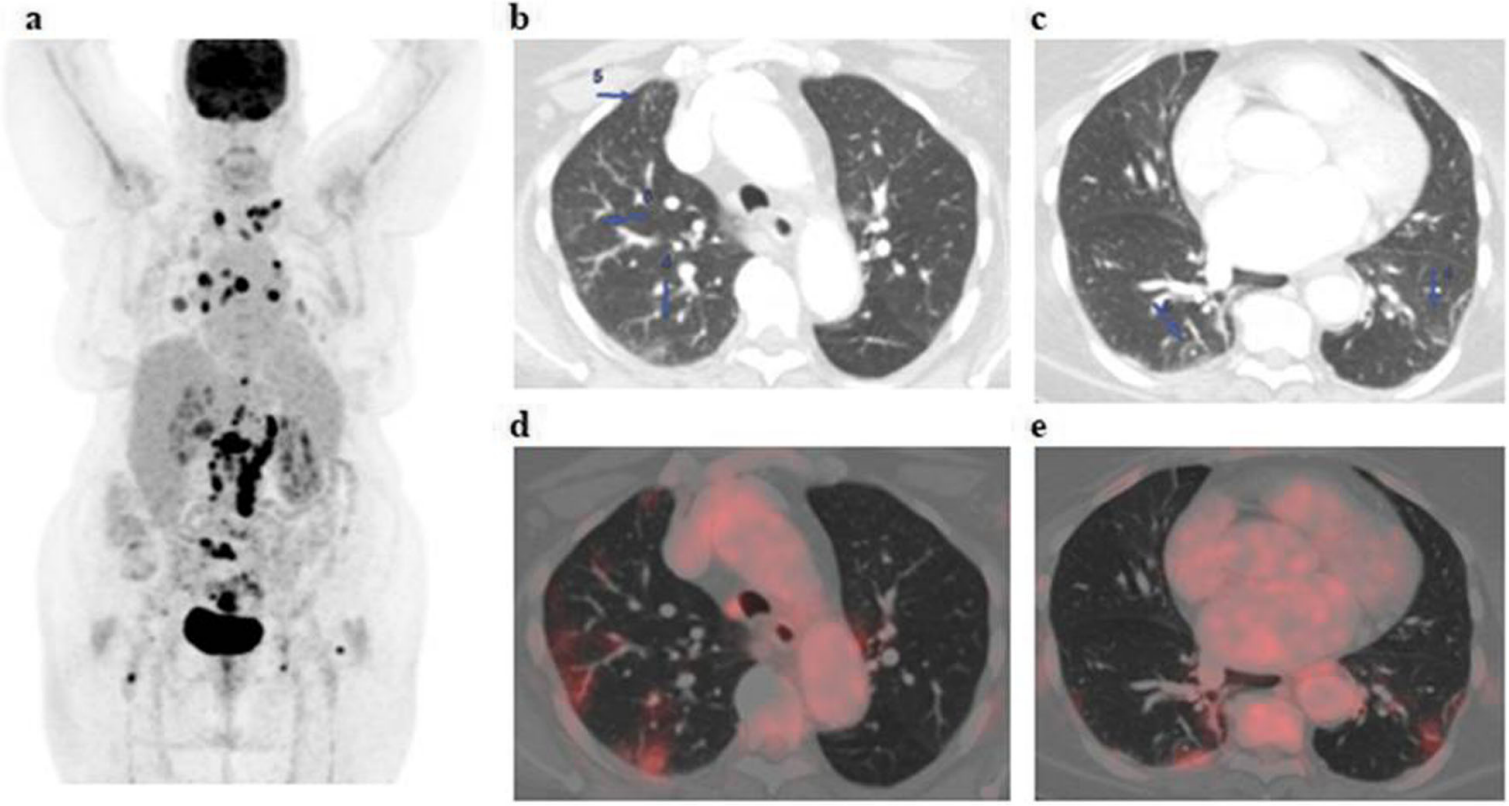
A 74-year-old female patient, presented with low back pain and previously done lumbosacral spine MRI which revealed malignant looking osseous lesions and referred for further assessment by PET/CT examination. The whole-body MIP image (**a**) shows multiple FDG avid nodal and osseous lesions with bilateral pulmonary areas of mild active FDG uptake. Axial lung window CT images (**b**, **c**) revealed small-sized bilateral subpleural ground-glass opacities that show mild FDG avidity on corresponding fused PET/CT images (**d**, **e**) achieving up to 4.3 SUVmax

**Fig. 4:**
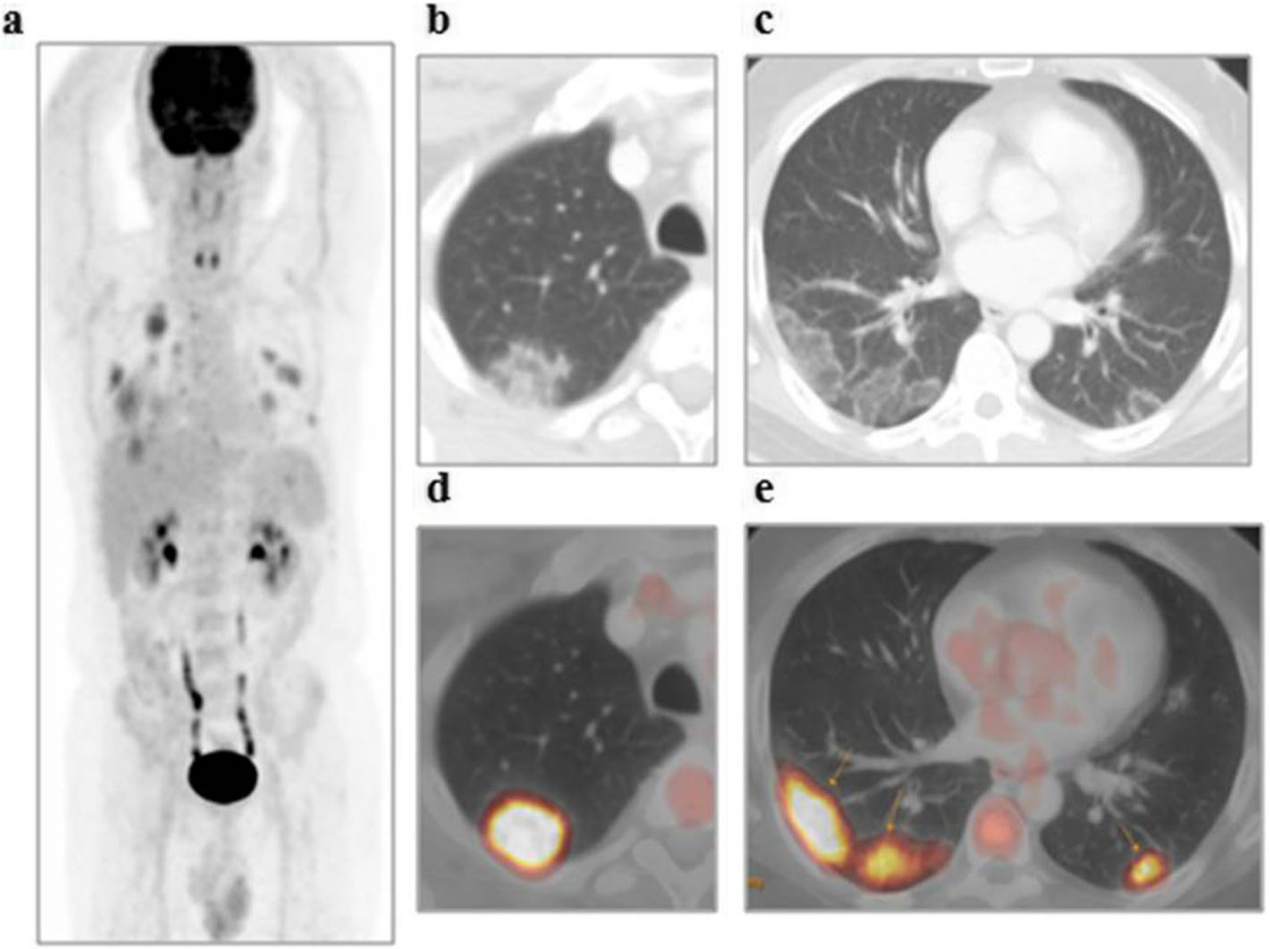
A 51-year-old male patient, with a history of treated lymphoma 5 years ago, referred for follow-up PET/CT evaluation. **a** The whole-body MIP image shows multiple bilateral pulmonary metabolically active areas. Axial lung window CT images (**b**, **c**) revealed multiple bilateral subpleural variable-sized ground-glass opacities associated with septal thickening (crazy-paving pattern) that show variable FDG avidity on corresponding fused PET/CT images (**d**, **e**) achieving up to 7.4 SUVmax

**Fig. 5:**
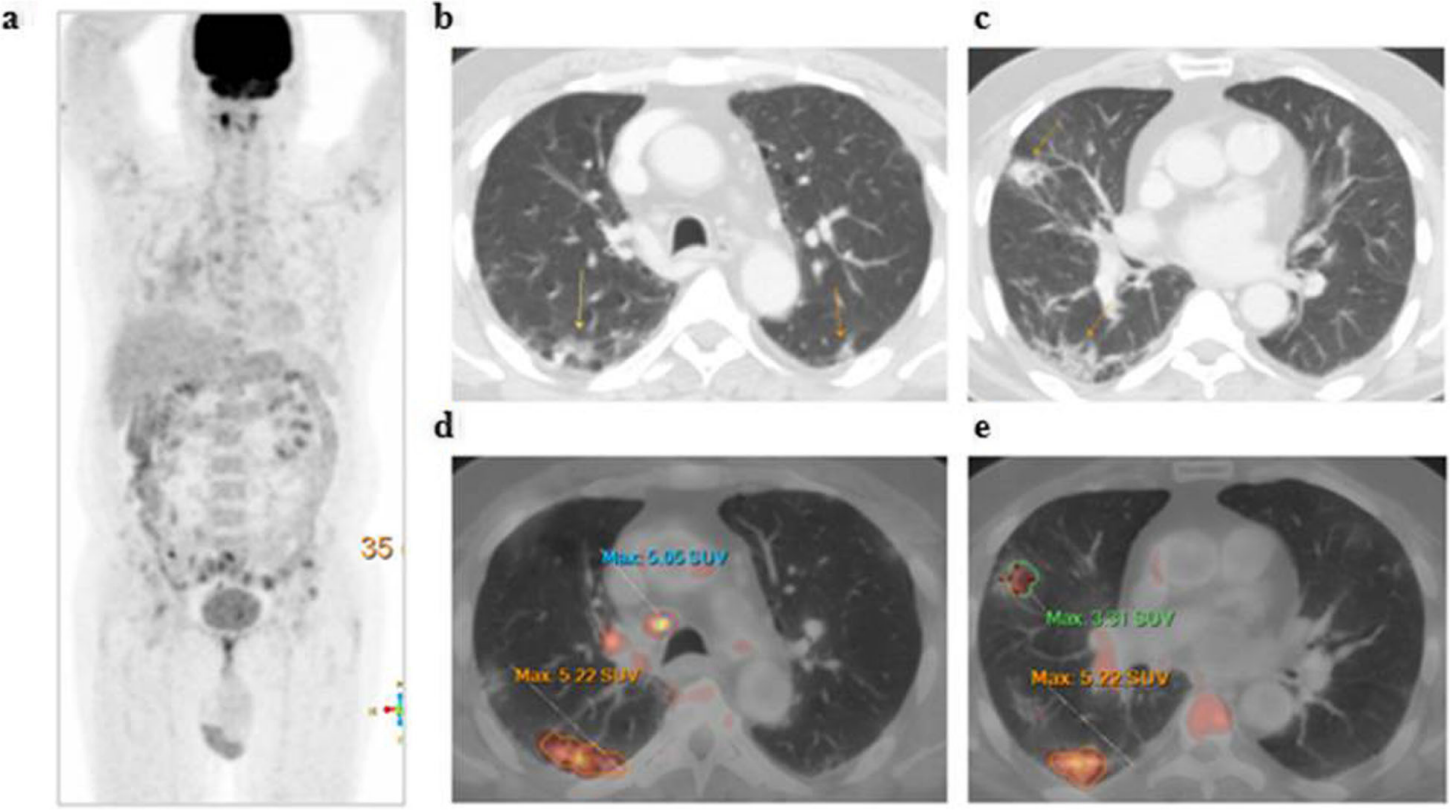
A 62-year-old male patient, with a history of surgically resected rectal cancer and referred for PET/CT follow-up. The whole-body MIP image (**a**) shows bilateral pulmonary metabolically active areas. Axial lung window CT images (**b**, **c**) revealed bilateral subpleural variable-sized ground-glass opacities with consolidation that show variable FDG avidity on corresponding fused PET/CT images (**d**, **e**) achieving up to 5.2 SUVmax

**Fig. 6:**
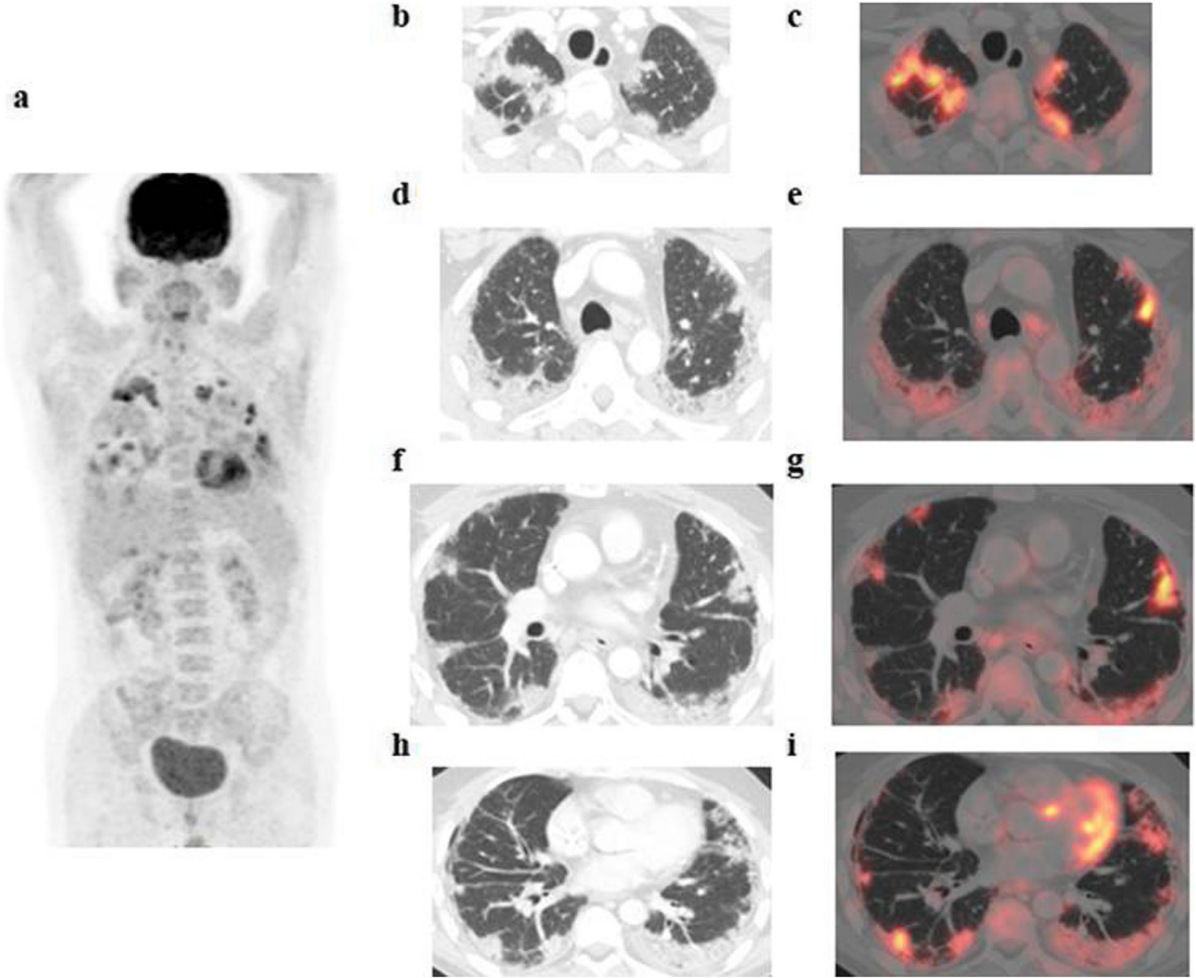
A 47-year-old male patient, with a history of left testicular seminoma, underwent left orchidectomy and received CTH, referred for follow-up PET/CT examination. The whole-body MIP image (**a**) shows bilateral multiple pulmonary metabolically active areas. Axial lung window CT images (**b**, **d**, **f**, **h**) revealed multiple bilateral subpleural variable-sized consolidations and ground-glass opacities with crazy-paving appearance that show variable FDG avidity on corresponding fused PET/CT images (**c**, **e**, **g**, **i**) achieving up to 10.9 SUV**max**

**Fig. 7:**
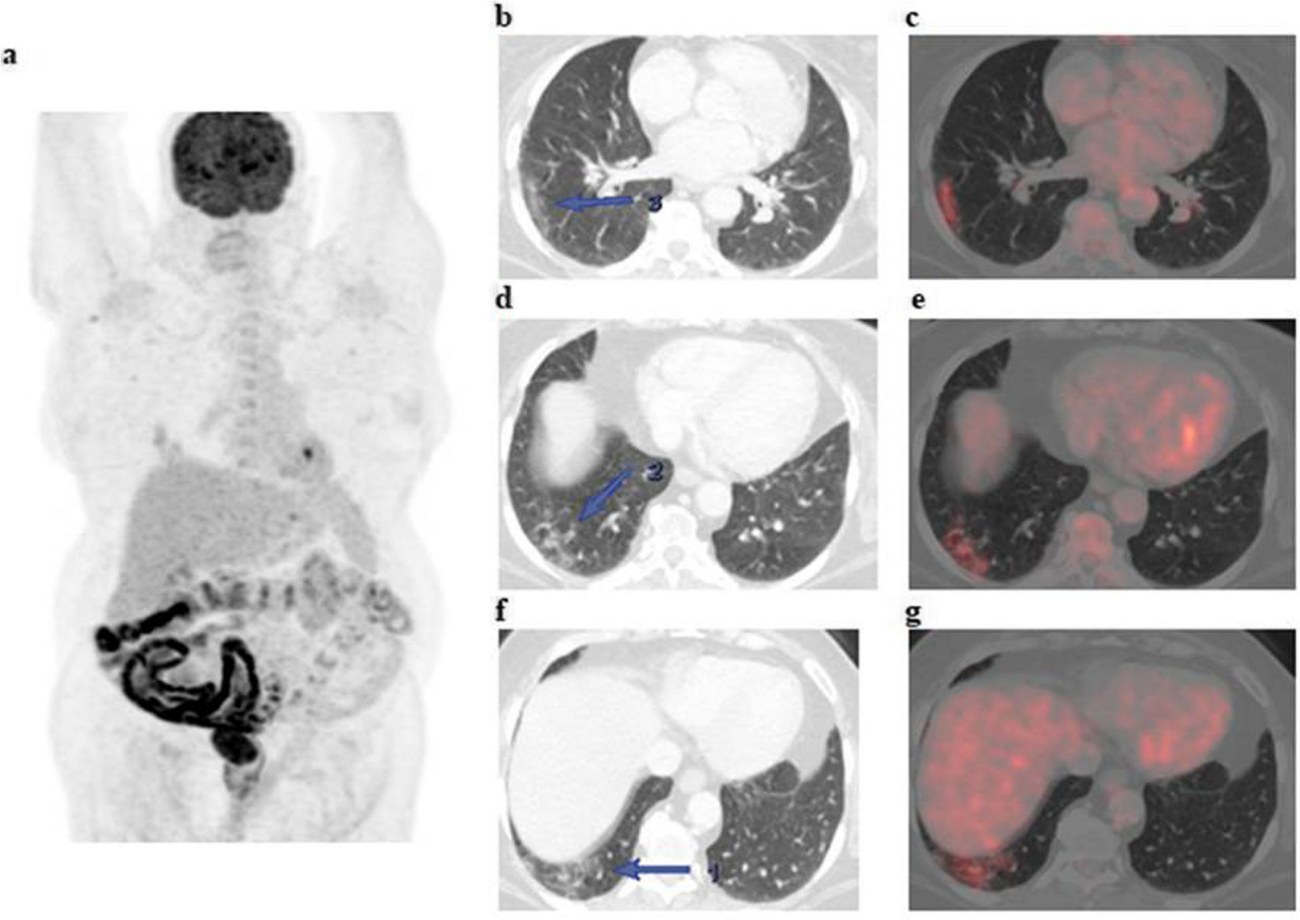
A 75-year-old female patient, with a history of lymphoma, received chemotherapy and referred for follow-up PET/CT evaluation. **a** The whole-body MIP image shows mildly active areas at the right lung lower zone. Axial lung window CT images (**b**, **d**, **f**) revealed few unilateral subpleural variable-sized ill-defined ground-glass opacities at the right lower lung lobe that show mild FDG uptake in corresponding fused PET/CT images (**c**, **e**, **g**) achieving up to 4.5 SUVmax

The patients (59 males and 28 females) ranged in age between 26 and 73 years with a mean age of about 53.5 ± 4.2 years. Thirty-two patients came for initial PET/CT assessment (Figs. 1, 2, and 3), and fifty-five patients came for follow-up PET/CT scans (Figs. 4, 5, 6, and 7). Thirty-two patients had lymphoma, twenty-six patients had breast cancer, fourteen patients had lung cancer, eleven patients had colon cancer, three patients had renal cancer, and one patient had ovarian cancer (Table 1).

**Table 1:** Demographic and imaging features of the study population

Characteristics	Number
**Age**	26 to 73 years (mean age of 53.5 ± 4.2 years)
**Gender**	
Males	59
Females	28
**Comorbidities**	
Lymphoma	32
Breast cancer	26
Lung cancer	14
Colon cancer	11
Renal cancer	3
Ovarian cancer	1
**Imaging features**	
**Lung affection**	
Unilateral	79
Bilateral	8
**Pneumonic pattern**	
GGOs	62
GGOs with consolidation patches	25
**SUVmax**	
4.3–7	46
6–9	27
9–12.6	14

The CT features suggesting COVID-19-associated pneumonia in the enrolled patients include multiple, mainly peripheral, ground-glass opacities and consolidations found bilaterally in 79 (90.8%) patients (Figs. 1, 2, 3, 4, 5, and 6) and unilaterally in 8 (9.2%) patients (Fig. 7). Pure ground-glass opacities were found in 62 (71.3%) patients, and mixed ground-glass opacities and consolidation patches were found in 25 (28.7%) patients. Intralobular reticulations were seen superimposed on the ground-glass opacities in 14 patients (16%), resulting in a crazy-paving pattern which is usually associated with more advanced stages of the disease (Fig. 4).

Mild pulmonary affection was found in 57 patients (65.5%), moderate pulmonary affection was found in 28 patients (32.2%), and severe pulmonary affection was found in 2 patients (2.3%).

On PET images, the pneumonic lesions show variable FDG avidity. The SUVmax ranged between 4.3 and 7 in 46 patients, between 7 and 9 in 27 patients, and between 9 and 12.6 in 14 patients. All the previous data is shown in Table 1.

**Fig. 8:**
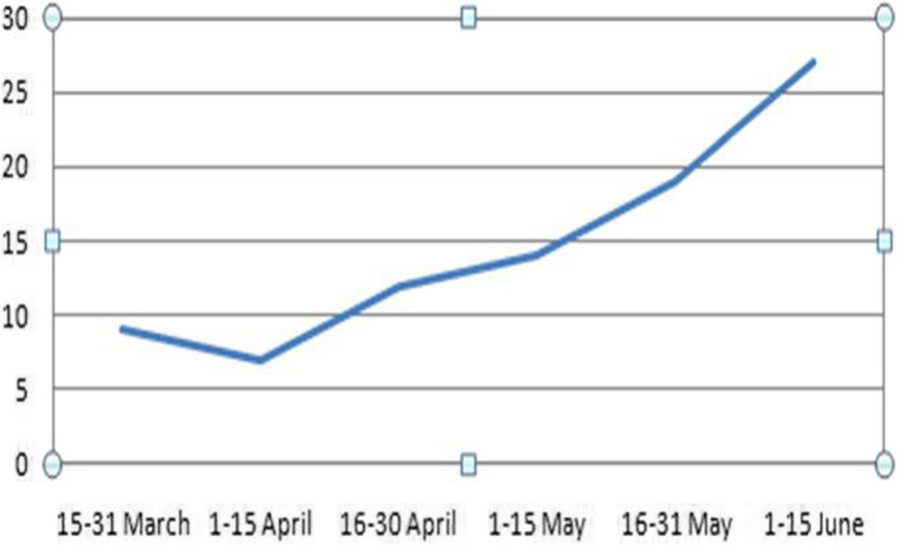
Line chart representation of the number of patients with recognized COVID-19-associated pneumonia in their routine oncologic 18F-FDG PET/CT examinations during the study period (the *Y* axis represents the number of cases, and the *X* axis represents the study time interval)

We detected 9 patients in the period from 15 to 31 March, 7 patients between 1 and 15 April, 12 patients between 16 and 30 April, 14 patients between 1 and 15 May, 19 patients between 16 and 31 May, and 26 patients between 1 and 15 June (Fig. 8).

**Fig. 9:**
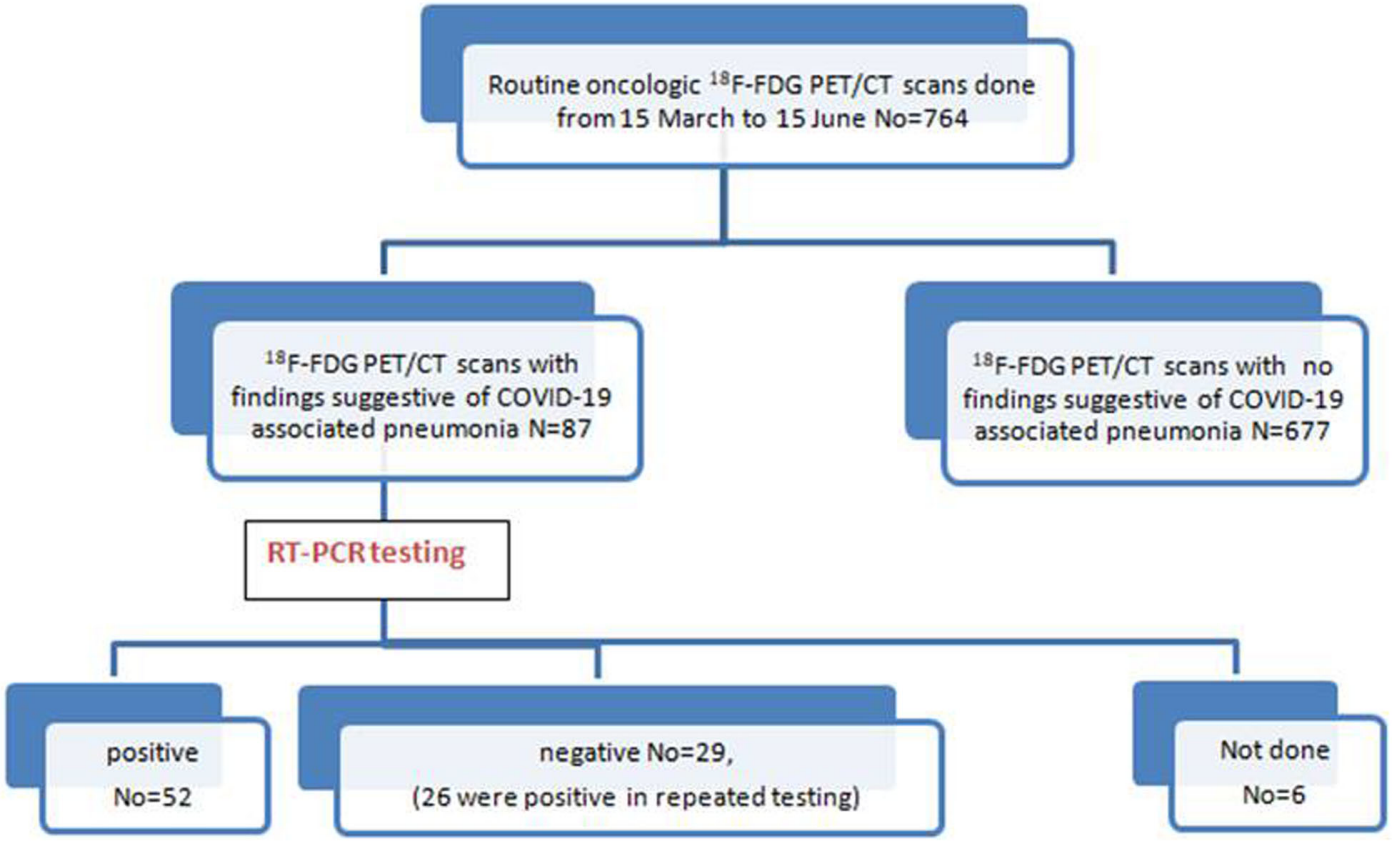
Flow chart representation of the research methodology and results

RT-PCR testing was done for confirmation of COVID-19 infection in 81 cases (Fig. 9) and was positive in 52 cases and negative in 29 cases that repeated it within 48 h, according to WHO recommendations, and turned positive in 26 cases (3 were still negative, yet they were isolated and carefully monitored for 2 weeks). In 6 of the 87 suspected patients, no RT-PCR testing was performed (was not mandatory in the absence of symptoms according to their physicians’ recommendations), yet they also underwent quarantine with careful monitoring for 2 weeks. Six of the closely monitored 9 patients became symptomatic few days after the study, and their physicians managed them as COVID-19-positive cases.

## Discussion

False negative results of nucleic acid testing (RT-PCR) to diagnose COVID-19 infection in substantial proportion of cases lead to significant missing of diagnosis especially in asymptomatic cases with more spread of the epidemic [12]. The Chinese authorities expanded the official definition of COVID-19 infection to include patients with typical CT findings, even with a first negative RT-PCR result [13]. Common CT findings of COVID-19-associated pneumonia have been widely reported and sometimes appeared even before the clinical symptoms become evident. Thus, high-resolution chest CT is considered a preferred tool for screening, diagnosis, severity assessment, and monitoring of COVID-19 pneumonia [14–16]. Early in the disease, single or multiple pulmonary subpleural nodular or segmental ground-glass opacities and consolidations were found that enlarged and spread centrally as the disease progressed. They may be accompanied by dilated pulmonary vasculature, air bronchogram sign, crazy-paving sign, reverse halo sign, interlobular septal thickening, pleural effusion, reactive LNs, and others. These CT features may be similar to those seen in other viral pneumonia such as MERS-CoV and SARS-CoV-2 [17].

^18^F-FDG PET/CT is a nonspecific nuclear imaging tool, used mainly for oncologic patients for the detection and staging of many malignant tumors [18–20]. It is also used for the follow-up of cancer patients [21–23]. ^18^F-FDG PET/CT is an efficient tool to assess response to treatment of different tumors [24, 25]. However, FDG uptake can be also seen in sites of active inflammation and infection [26]. Many recent case reports and studies reported findings of COVID-19 pneumonia in patients referred for routine nuclear imaging studies, including ^18^F-FDG PET/CT scans [27–29]. Typical CT features were detected in localizing CT that show increased FDG in corresponding PET images [30–32].

In this study, 87 of included 764 patients (about 11.4%) show incidental CT features of COVID-19-associated pneumonia that are FDG avid on the corresponding PET images (ranged between 4.3 and 12.6 SUVmax).

Our results matched with those reported by Albano et al. [33] and Setti et al. [34] who incidentally identified features highly suspicious for COVID-19 pneumonia in asymptomatic Italian cancer patients coming for routine ^18^F-FDG PET/CT examinations. Albano et al. detected typical CT features in 6 of 65 patients (9%), 4 patients had positive RT-PCR test and the remaining 2 patients were not tested but underwent quarantine and monitoring. Setti et al. found typical CT features in 5 out of 13 patients with SUVmax ranged between 4.3 and 11.3.

Pellardy et al. detected CT abnormalities in 3.8% of asymptomatic cancer patients referred to a French PET center for routine oncological indication in the period from March 2020 to April 2020 and ^18^F-FDG avidity was ranged between 1.58 and 13.64 [35].

This confirmed the possibility to have COVID-19 pneumonia without symptoms which increase the risk of spread of infection. Also, it emphasizes the importance of applying hygienic measures, optimizing distance, minimizing contact, and using protective equipment during this pandemic for the safety of both health care personnel and the patients (as cancer patients particularly those under chemotherapy are more susceptible for a severe infection) [36]. On the other hand, recognition and reporting of COVID-19-related findings to the referring physicians is important for immediate and appropriate action to be undertaken as home isolation with adequate clinical monitoring and postponing oncological treatments that are potentially affecting the patient’s survival [33].

Other studies had suggested the potential role of ^18^F-FDG PET/CT scan in monitoring of treatment response and predicting the recovery time in COVID-19 cases. The higher the FDG uptake in the pulmonary lesions, the longer healing times [32, 37]. However, these are obviously just case observations, which need to be properly characterized in larger patient cohorts before conclusions can be drawn.

Moreover, whole-body ^18^F-FDG PET/CT scan may be of value to evaluate other changes elsewhere in the patient’s body as damage to other organs such as the gastrointestinal tract, heart, kidneys, or bone marrow can also occur in COVID-19 infection [38–40].

The main limitation of our study was the small sample size, and future multicenter studies with larger number of enrolled patients are recommended to obtain more accurate results.

## Conclusion

^18^F-FDG PET/CT is sensitive for early COVID-19 detection, even in asymptomatic patients which in turn contribute to proper management and minimize the spread of hidden infection. It also highlights the key role of the radiologist in the identification of imaging findings suggestive of the disease in this pandemic era and emphasizes the importance of following safety measures and the use of appropriate personal protective equipment to minimize COVID-19 transmission among the patients and health care givers.

## Data Availability

All data generated or analyzed during this study are included in this article.

## Abbreviations

MERS-CoV: Middle East respiratory syndrome coronavirus
SARS-CoV-2: Severe acute respiratory syndrome coronavirus 2
COVID-19: Coronavirus disease 2019
PET/CT: Positron emission tomography/computed tomography
^18^F-FDG: ^18^F-Fluorodeoxyglucose
RT-PCR: Reverse transcriptase-polymerase chain reaction
HU: Hounsfield units
MIP: Maximum intensity projection
GGOs: Ground-glass opacities
WHO: World Health Organization
